# Generation of 1,2-azaboretidines *via* reduction of ADC borane adducts†
†Electronic supplementary information (ESI) available: Experimental, crystallographic, and computational details. CCDC 1042591–1042594. For ESI and crystallographic data in CIF or other electronic format see DOI: 10.1039/c5sc01077b



**DOI:** 10.1039/c5sc01077b

**Published:** 2015-04-16

**Authors:** H. Braunschweig, A. Gackstatter, T. Kupfer, T. Scheller, F. Hupp, A. Damme, N. Arnold, W. C. Ewing

**Affiliations:** a Institut für Anorganische Chemie , Julius-Maximilians-Universität Würzburg , Am Hubland , 97074 Würzburg , Germany . Email: h.braunschweig@uni-wuerzburg.de ; http://www-anorganik.chemie.uni-wuerzburg.de/Braunschweig/

## Abstract

Reaction of the acyclic (diamino)carbene (ADC) :C(NiPr_2_)_2_ (**1**) with different dihaloboranes of the type RBX_2_ (R = Mes, Dur; X = Cl, Br) smoothly afforded a novel class of ADC-stabilized borane adducts. For MesBBr_2_ however, the reaction did not stop at the adduct level, but an uncommon rearrangement process occurred, which eventually resulted in the formation of a 5-membered boracycle after elimination of mesitylene. Chemical reduction of the ADC borane adducts by KC_8_ selectively yielded air stable 1,2-azaboretidines. Detailed DFT studies suggest a reduction mechanism involving a highly reactive borylene intermediate, which is converted into the boracycles *via* a rearrangement/C–H activation sequence.

## Abstract 2

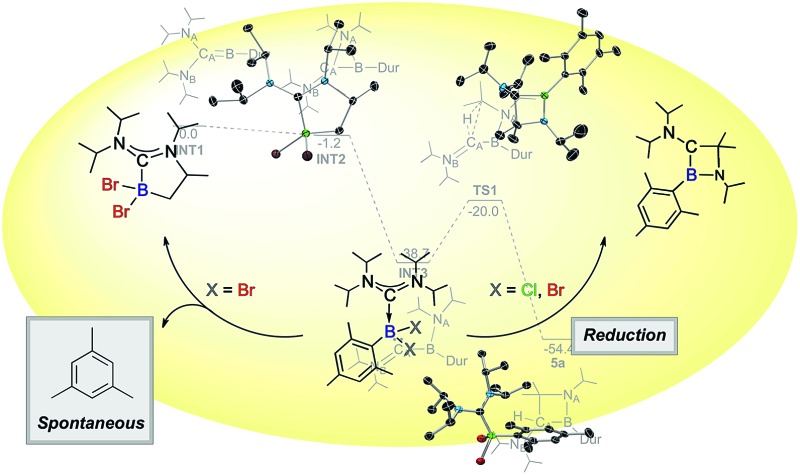
ADC borane adducts RBX_2_·ADC (R = Mes, Dur; X = Cl, Br; ADC = :C(NiPr_2_)_2_) have been prepared and reduced by KC_8_ to afford air stable 1,2-azaboretidines with high selectivity.

## Introduction

Recent years have clearly pointed out the exceptional value of carbenes not only as ligands in catalysis,^1^ but also in the stabilization of highly reactive transition metal and main group element species.^2^ Initially, research focused on the use of *N*-heterocyclic carbenes (**A**; NHC) as stabilizing Lewis bases, and from 2007 a series of outstanding molecules including diborenes,^3^ diatomic allotropes (B_2_,^4^ Si_2_,^5^ Ge_2_,^6^ Sn_2_,^7^ P_2_,^8^ As_2_^9^), and radical/radical ions (B, Si, pnictogen, organic)^2*b*^ could be realized. Later on, it was rapidly recognized that the different electronics of cyclic (alkyl)(amino)carbenes (**B**; CAAC) can also be quite useful in the generation of such species (*e.g.* B_2_,^10^ P_2_,^11^ PN,^12^ Sb_2_,^13^ B/Si/P/Sn radicals).^2*e*^ In many cases however, CAAC stabilization also significantly alters the reactivity of the adduct precursors, thereby granting access to other classes of products.^2*e*^

The impact of the subtle electronic differences between NHC and CAAC donor ligands is maybe best illustrated by the reduction chemistry of carbene-stabilized haloboranes. Thus, we have demonstrated that chemical reduction of NHC adducts of the type RBX_2_·NHC and B_2_Br_4_·NHC_2_ is associated with the generation of boron multiple-bonded species, *i.e.* diborenes^3^ and diborynes.^4^ By contrast, reduction of DurBCl_2_·CAAC (Dur = 2,3,5,6-tetramethylphenyl) stops at the radical stage enabling the isolation of a neutral boron-containing radical, which is most likely a direct consequence of the stronger π accepting abilities of the CAAC ligand.^14^ This particular electronic property of the CAAC ligand is also reflected in the cumulenic structure of B_2_·CAAC_2_, which shows a decreased B–B bond order in comparison to that of the triply bound B_2_·NHC_2_.^15^ Fascinating results also come from the group of Bertrand, which highlighted that CAACs efficiently stabilize neutral B–H borylene^16^ and boryl radical species,^17^ as well as boryl anions.^18^ 
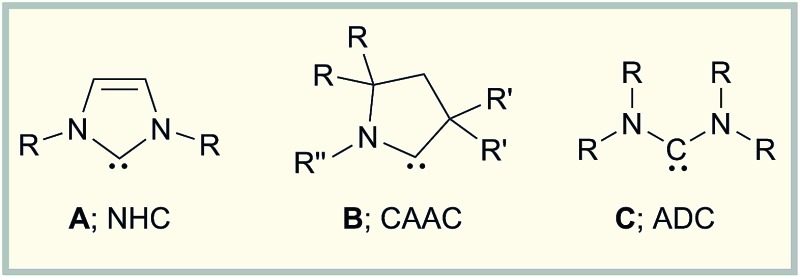


With these developments in mind, we wondered why the potential of acyclic (diamino)carbenes (**C**; ADC) in the stabilization of uncommon low-valent main group element compounds has not been studied in detail so far. This appears particularly surprising given the remarkable structural and electronic properties of ADCs.^19^ Thus, the wider N–C–N angle in ADCs increases the steric shielding of the carbene carbon atom by the amino substituents, which will also affect the structural environment of any potential bonding partner in providing a larger degree of steric protection to the reactive center.^20^ In addition, ADCs offer a significantly higher basicity, nucleophilicity, and σ donor capacity than NHCs,^19^ which might entail other reactivity patterns for the reduction of suitable ADC adducts than those observed for their NHC/CAAC analogs. So far however, ADCs have predominately found application as flexible ligands in transition metal catalysis,^19^ and the number of publications addressing its main group element chemistry is rather limited. Sporadic studies deal with coupling reactions, decomposition reactions, or small molecule activation,^19a,21^ while almost nothing is known about their ability to stabilize reactive main group element compounds.

As part of our ongoing efforts to generate uncommon low-valent boron species, we now began to study the chemistry of simple ADC borane adducts of the type RBX_2_·ADC with an emphasis on their reduction behavior, and the results are presented in this contribution.

## Results and discussion

**Scheme 1:**
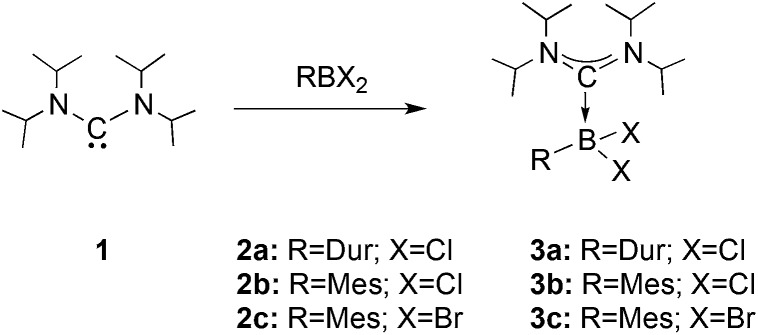
Syntheses of ADC borane adducts **3a–c**.

**Fig. 1:**
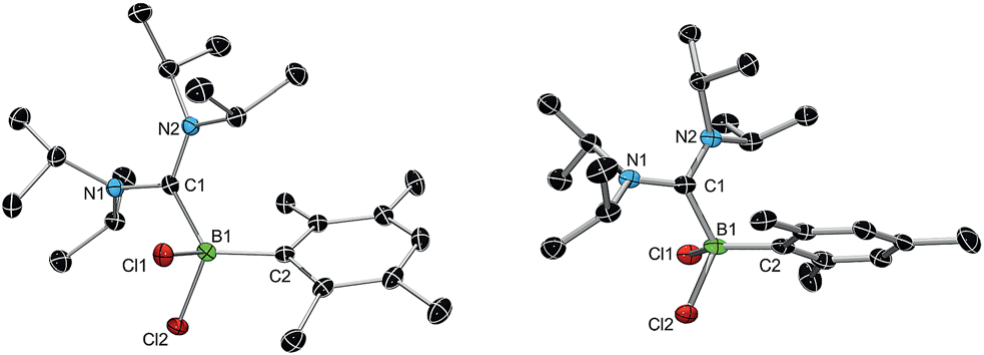
X-ray diffraction structures of **3a** (left) and **3b** (right). Hydrogen atoms and co-crystallized solvent molecules are omitted for clarity. Selected bond lengths [Å] and angles [°]: **3a**: B1–C1 1.681(3), B1–C2 1.622(2), B1–Cl1 1.938(2), B1–Cl2 1.889(2), C1–N1 1.376(2), C1–N2 1.352(2), C1–B1–C2 118.9(1), Cl1–B1–Cl2 103.71(9), N1–C1–N2 117.0(2); **3b**: B1–C1 1.676(5), B1–C2 1.623(5), B1–Cl1 1.910(4), B1–Cl2 1.920(4), C1–N1 1.364(5), C1–N2 1.365(4), C1–B1–C2 113.5(3), Cl1–B1–Cl2 101.4(2), N1–B1–N2 116.6(3).

We chose bis(diisopropylamino)carbene **1** as the most suitable ADC for the realization of ADC borane adducts, because of its high stability and its convenient synthetic access. **1** was first prepared by Alders *et al.* back in 1996 as the first compound of this class, and can be readily isolated in high yields by sublimation.^22^ The stoichiometric reactions of **1** with different dihaloboranes RBX_2_ (**2a**: R = Dur, X = Cl; **2b**: R = Mes, X = Cl; **2c**: R = Mes, X = Br; Mes = 2,4,6-trimethylphenyl) proceeded spontaneously and with high selectivity to afford ADC borane adducts **3a–c** in almost quantitative yields (**3a**: 92%; **3b**: 93%; **3c**: 97%; Scheme 1). Identification of **3a–c** posed no difficulties, and ^11^B NMR spectroscopy in solution (**3a**: *δ* = 4.8; **3b**: *δ* = 4.5; **3c**: *δ* = –1.3) and X-ray diffraction studies on **3a** and **3b** (Fig. 1) clearly confirmed adduct formation, and the presence of tetra-coordinate boron centers. The solid-state structures of **3a** and **3b** revealed no surprises, and all bonding parameters are reminiscent of other known carbene borane adducts. As anticipated, both structures feature N1–C1–N2 bond angles (**3a**: 117.0(1)°; **3b**: 117.1(4)°) that are smaller than in **1** (121.0(5)°).^22^

**Scheme 2:**
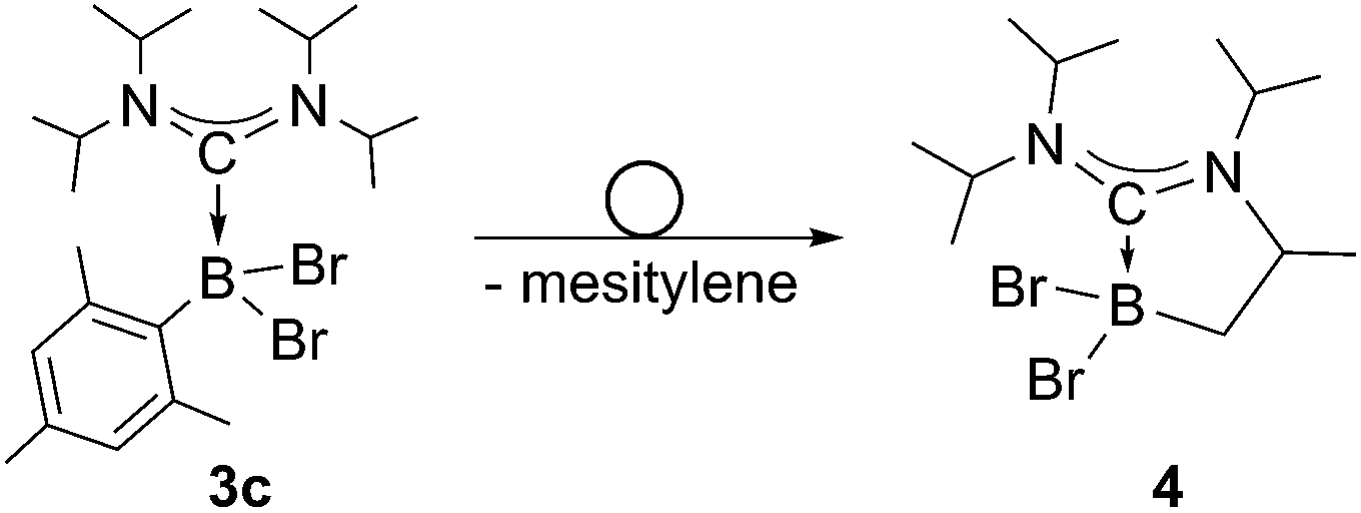
Rearrangement of ADC borane adduct **3c**.

**Fig. 2:**
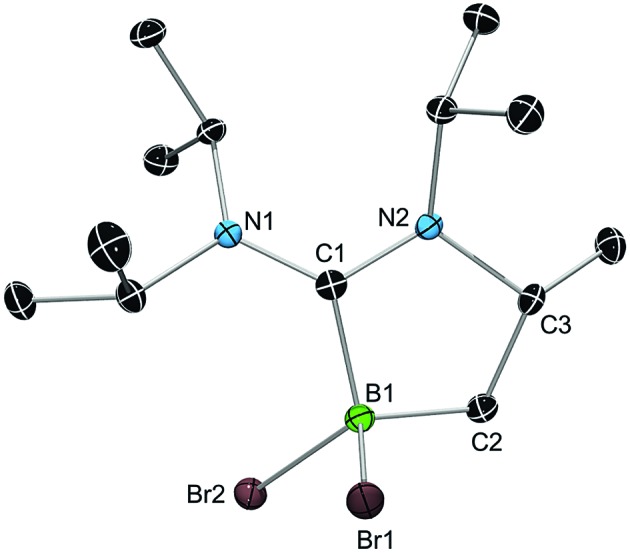
X-ray diffraction structure of **4**. Hydrogen atoms are omitted for clarity. Selected bond lengths [Å] and angles [°]: B1–C1 1.624(2), B1–C2 1.598(3), B1–Br1 2.049(2), B1–Br2 2.067(2), C1–N1 1.352(2), C1–N2 1.340(2), N2–C3 1.507(2), C2–C3 1.532(2), C1–B1–C2 99.53(1), B1–C2–C3 104.3(1), C2–C3–N2 104.5(1), C3–N2–C1 113.3(1), N2–C1–B1 109.0(2), N1–C1–N2 122.8(2).

While **3a** and **3b** were found indefinitely stable under inert conditions, **3c** readily undergoes a rearrangement reaction to afford boracycle **4** (Scheme 2). The process occurs both in solution (24 h), and in the solid-state (4–5 days), and is quantitative as judged by ^1^H NMR and ^11^B NMR spectroscopy (**3c**: *δ* = –1.3; **4**: *δ* = –3.4). From a mechanistic point of view, rearrangement of **3c** involves C–H activation of one iPr group with concomitant elimination of mesitylene to generate the 5-membered heterocycle **4**, whose identity was eventually verified by an X-ray diffraction study (Fig. 2). As a consequence of ring formation, the N1–C1–N2 angle (122.8(2)°) in **4** is slightly larger than in adducts **3a** (117.0(1)°) and **3b** (117.1(4)°), while the C1–B1–C2 angle (99.53(1)°) becomes dramatically smaller (**3a**: 118.9(1)°; **3b**: 113.5(3)°). By contrast, the C1–N1 (1.352(2) Å) and C1–N2 (1.340(2) Å) bond lengths remain almost unaffected (*cf.* **3a**: 1.376(2) Å, 1.352(2) Å; **3b**: 1.364(5) Å, 1.365(4) Å), which suggests that the carbenic character of C1 is retained upon rearrangement. The elimination of mesitylene induced by the coordination of the ADC to the boron center of **3c** is highly unexpected and should definitely be emphasized here. To the best of our knowledge, such a behavior has not been observed before in boron chemistry. All cases that report on somekind of rearrangement/elimination processes upon coordination of a σ donor to a haloborane were accompanied exclusively by HX elimination (X = Br, I), which usually seems to be strongly favored over RH elimination.^23^

**Scheme 3:**
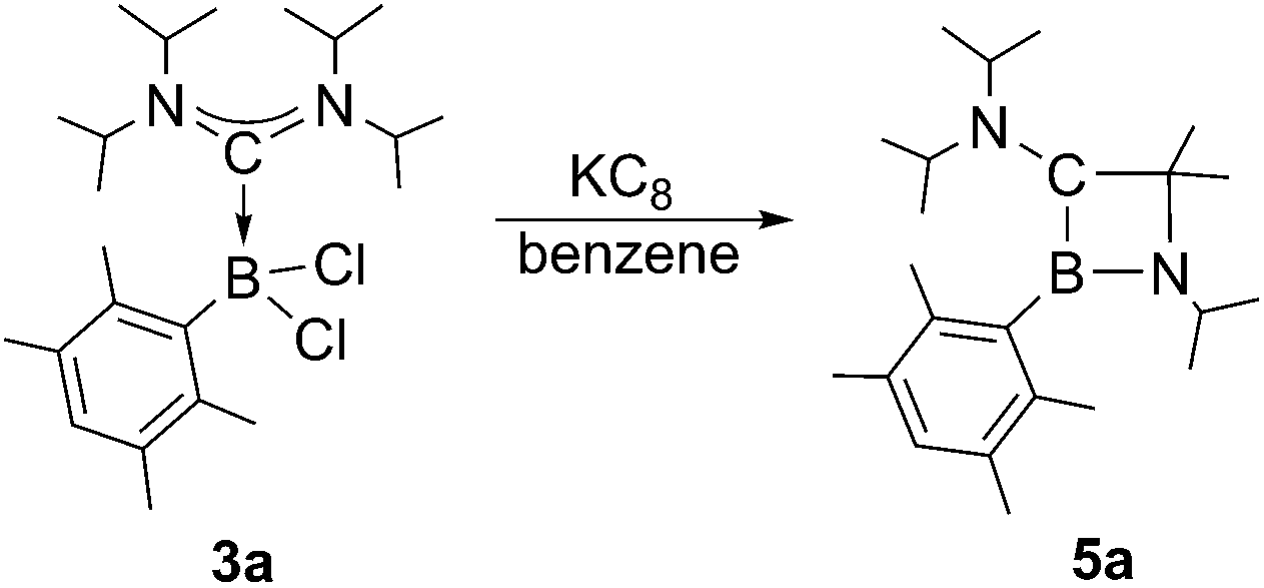
Synthesis of 1,2-azaboretidine **5a**.

Next, we studied the reduction chemistry of ADC borane adducts **3a–c**. To this end, **3a** was treated with an excess of KC_8_ in benzene solution (Scheme 3). Initially, the reaction mixture turned red in color, which however completely disappeared within one hour to finally leave a colorless solution. ^11^B NMR spectroscopy indicated quantitative and selective conversion of **3a** into a new boron-containing species (**5a**: *δ* = 45.0). After work-up, 1,2-azaboretidine **5a** was isolated as air stable, colorless crystals by recrystallization from hexanes.

**Fig. 3:**
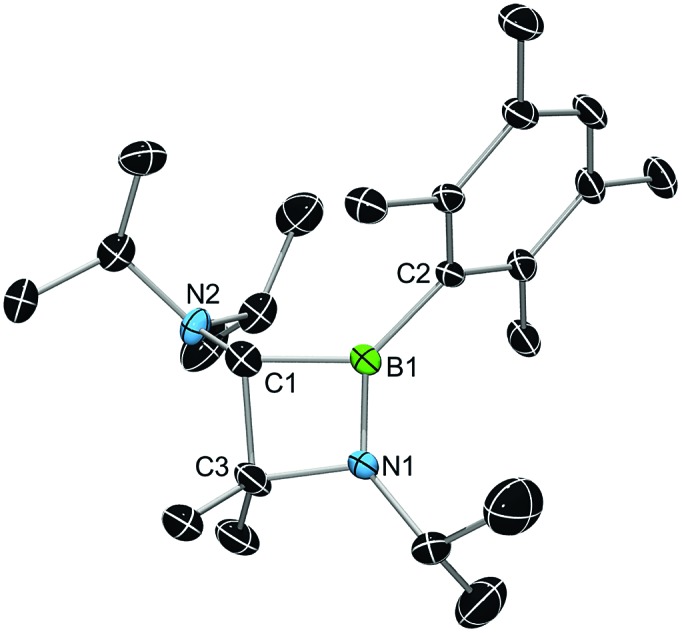
X-ray diffraction structure of **5a**. Hydrogen atoms are omitted for clarity. Selected bond lengths [Å] and angles [°]: B1–C1 1.624(4), B1–C2 1.577(4), B1–N1 1.398(4), C1–N2 1.446(4), C1–C3 1.599(5), C3–N1 1.498(4); C1–B1–N1 91.1(2), B1–N1–C3 96.0(2), N1–C3–C1 88.5(2), C3–C1–B1 83.8(2), B1–C1–N2 128.0(3).

X-ray diffraction served to validate the molecular composition of **5a** in the solid-state (Fig. 3). Accordingly, reduction of **3a** is followed by a 1,2-shift of one NiPr_2_ moiety from the carbenic carbon C1 to the boron center, and a subsequent C–H activation/ring closure sequence to afford the 4-membered heterocycle **5a**. The structural parameters of **5a** are fully consistent with a classification as 1,2-azaboretidine, and relevant bond lengths and angles lie within the same range observed for other 1,2-azaboretidines.^24^ Thus, the boron center adopts a distorted trigonal planar geometry. The C1–B1–N1 angle of 91.1(2)° indicates the presence of an almost regular BC_2_N rectangle (B1–N1–C3: 96.0(2)°; N1–C3–C1: 88.5(2)°; C3–C1–B1: 83.8(2)°), which however is not completely planar (torsion angles between –5.0(2)° and 4.9(2)°). Due to the C–H activation event involving the carbene center of **3a**, the geometry of C1 changed from trigonal planar in **3a** to highly distorted tetrahedral in **5a** (B1–C1–N2: 128.0(3)°).

The use of ADC **1** has been previously implicated in the formation of 4-membered rings in its reaction with CO, wherein initial complexation of CO and resultant ketene formation leads to the migration of one of the two ADC NiPr_2_ groups across the C=C bond, initially forming an amide complex that subsequently cyclizes to the final β-lactam product.^21b,25^ A similar mechanism can be invoked to explain the formation of the 1,2-azaboretidine, since the initial reduction of **3a** presumably yields a high-energy borylene (ADC = B–Dur), analogous to the aforementioned ketene (ADC = C=O).

**Fig. 4:**
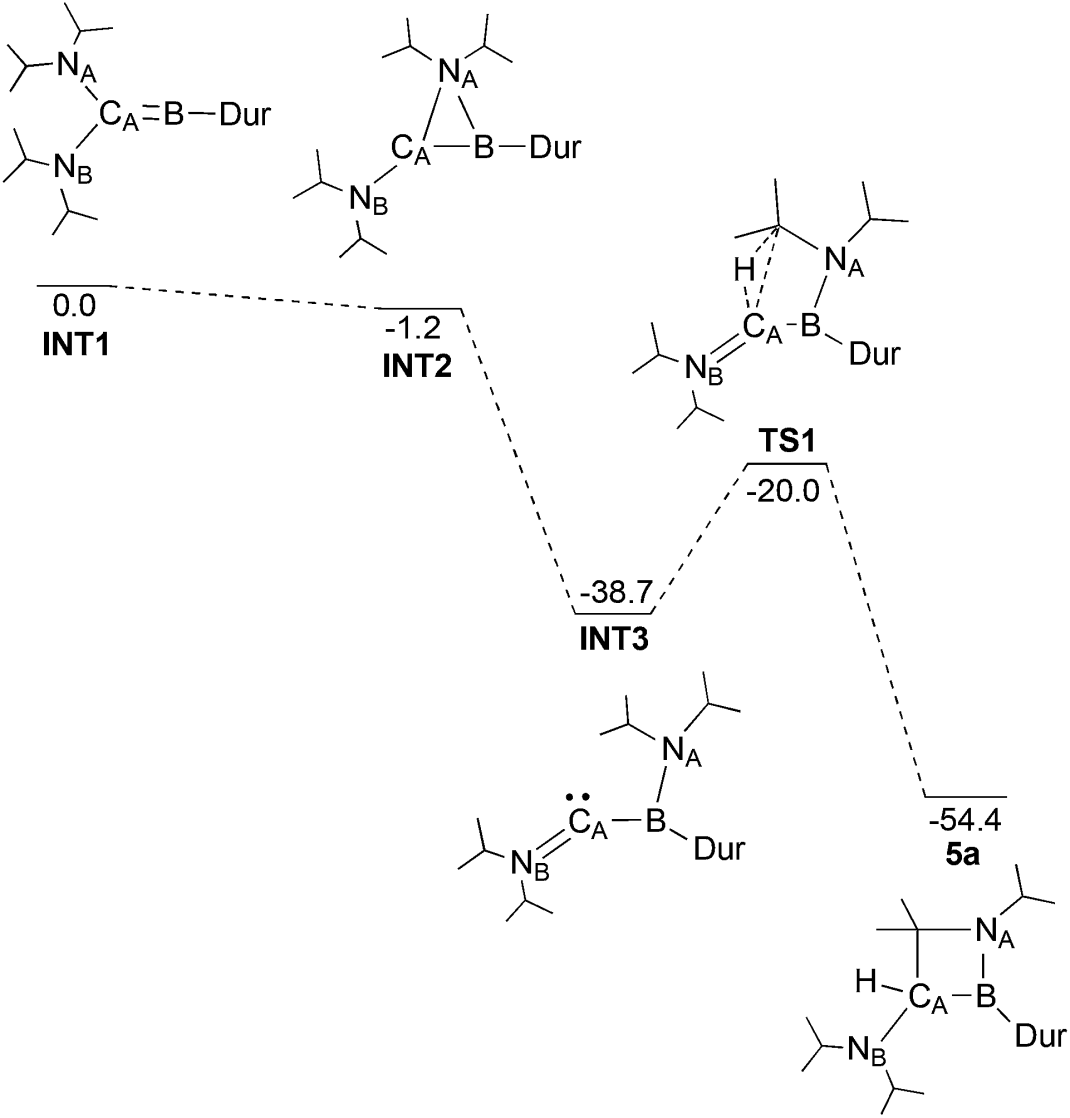
Proposed reaction pathway from the initial borylene product of the reduction of **3a** to **5a**, as calculated by DFT (B3LYP/6-311G(d)). Electronic energies are given in kcal mol^–1^ and are ZPE-corrected.

Through DFT calculations (B3LYP/6-311G(d)) we were able to investigate such a mechanism (Fig. 4). A structure for the post-reduction high energy borylene was successfully identified (**INT1**), showing a linear C_A_–B–C_Dur_ unit (175.0°), as well as one wide and one more acute N–C–B angle (N_A_–C_A_–B: 106.8°; N_B_–C_A_–B: 134.3°). The transition states in the migration of the diisopropylamine could not be located, likely as a result of the shallow potential energy surface in this region, but an intermediate was found with N_A_ bridging C_A_ and B (**INT2**) only 1.2 kcal mol^–1^ lower in energy than **INT1**. Completion of the migration of the diisopropylamine group from C_A_ to B (**INT3**) results in a large decrease in energy (–37.5 kcal mol^–1^), giving a structure with a planar boron (*Σ*_angles_ = 359.5°), but a pronounced bend at C_A_ (B–C_A_–N_B_: 143.4°) and a short C_A_–N_B_ bond (1.282 Å). Activation of the central C–H bond on one of the two iPr groups on N_A_ presumably leads to **TS1**, 18.7 kcal mol^–1^ higher in energy than **INT3**, but still 20.0 kcal mol^–1^ lower in energy than the initial borylene. Given the high activation barrier for this rearrangement process however, a mechanism involving boron-based radical intermediates might also be effective at this stage, even though we did not obtain any definite experimental evidence for the presence of such radical species. Completion of the C–H activation gives compound **5a**, 54.4 kcal mol^–1^ lower in energy than the borylene product of the reduction of **3a**.

**Scheme 4:**
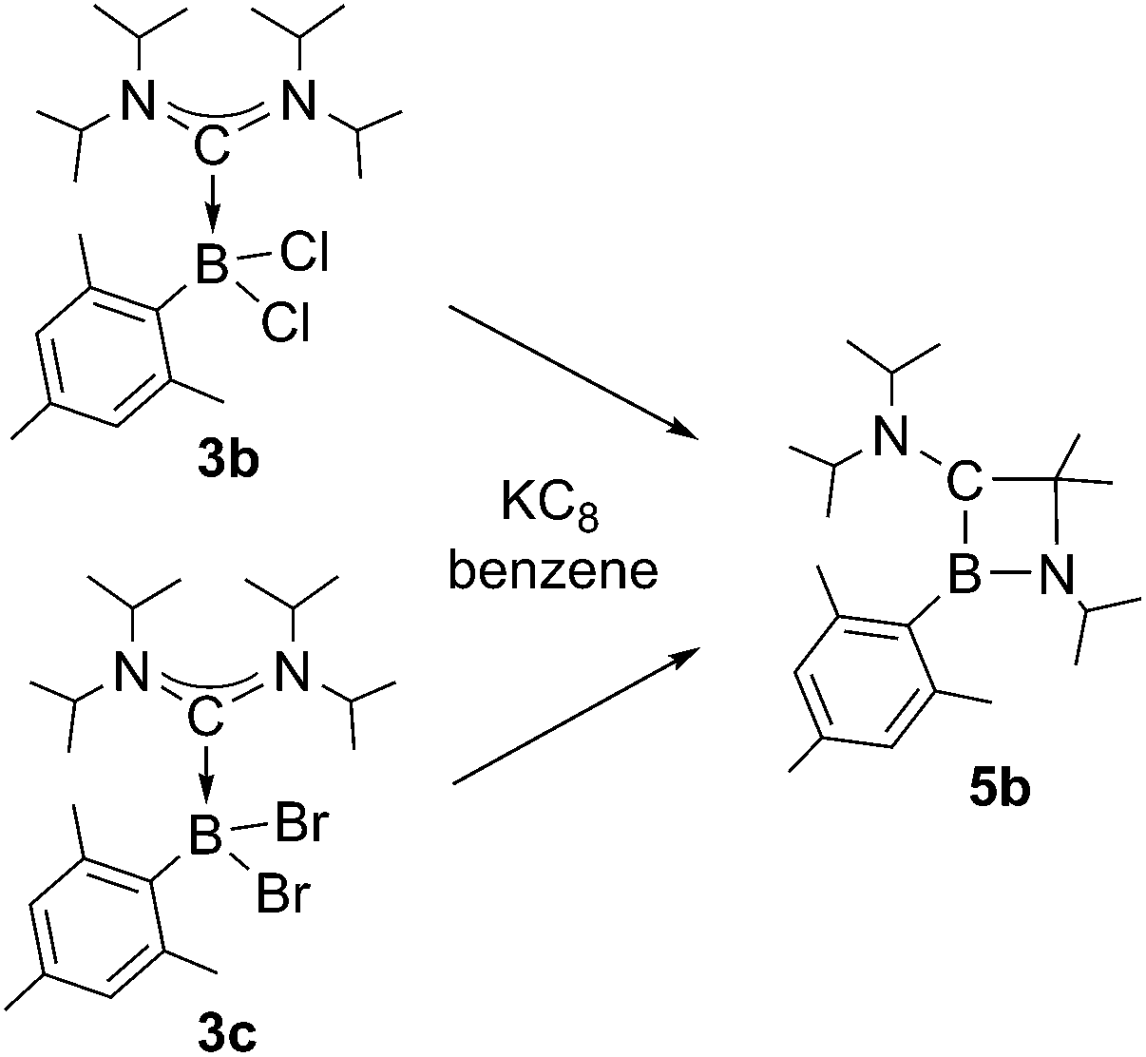
Synthesis of 1,2-azaboretidine **5a**.

Chemical reduction of ADC borane adducts **3b** and **3c** pursues an identical reaction pathway as observed for **3a** (Scheme 4), and in both cases 1,2-azaboretidine **5b** is formed with high selectivity as suggested by ^11^B NMR spectroscopy of the reaction mixtures (*δ* = 44.9). **5b** can only be isolated as an colorless oil after the standard work-up procedure, which precluded a determination of its molecular structure by X-ray diffraction. The results of NMR spectroscopic studies and an elemental analysis however clearly legitimate the assigned composition.

## Conclusions

In summary, we have demonstrated that the ADC borane adducts **3a–c** are conveniently accessible by stoichiometric reaction of dihaloboranes RBX_2_ with bis(diisopropylamino)-carbene **1**. While the chloro-substituted species **3a** and **3b** were found indefinitely stable under ambient conditions, the bromo-substituted derivative **3c** was prone to undergo a rearrangement process both in solution, and in the solid-state to afford the 5-membered heterocycle **4** *via* C–H activation and mesitylene elimination steps. Subsequent chemical reduction of the ADC borane adducts **3a–c** led to the formation of air stable 1,2-azaboretidines **5a** and **5b**. According to DFT calculations, this transformation most likely involves the initial formation of a reactive borylene species, which is converted into the heterocyclic products by a sequence of 1,2-NiPr_2_-shift and C–H activation/ring closure.
