# Supplementary material for: Generation of 1,2-azaboretidines via reduction of ADC borane adducts
Source: Chem Sci. 2015 Apr 16;6(6):3461–5. doi: 10.1039/c5sc01077b (PMC5492900; doi:10.1039/c5sc01077b)
Supplement: Supplementary file 1 [file SC-006-C5SC01077B-s001.pdf]

Electronic Supplementary Material (ESI) for Chemical Science.
This journal is © The Royal Society of Chemistry 2015

Electronic Supplementary Material (ESI) for *Chemical Science*.

Generation of 1,2-Azaboretidines *via* Reduction of ADC Borane Adducts.

H. Braunschweig,*[a] A. Gackstatter, T. Kupfer, F. Hupp, T. Scheller, A. Damme, N. Arnold and W. C. Ewing

Experimental Details

General Considerations:

All manipulations were performed under a dry argon atmosphere in an INNOVATIVE TECHNOLOGY glove box or using standard Schlenk line techniques. All solvents were dried using standard methods, and stored over molecular sieves after distillation under argon. Bis(di*iso*propylamino)carbene (**1**),[1] $DurBCl_2$,[2] $MesBCl_2$,[3] and $MesBBr_2$[4] were prepared according to literature procedures. NMR spectra were measured on a Bruker Avance 400, or a Bruker Avance 500 FT-NMR spectrometer. ^{11}B NMR spectra were referenced to external $BF_3•OEt_2$. ^1H NMR and $^{13}C\{^1H\}$ NMR spectra were referenced to external TMS *via* the residual protons of the solvent (^1H), or the solvent itself (^{13}C). The chemical shift δ is reported in ppm. Elemental analyses were performed on a Carlo Erba model 1106, or a Leco instruments CHNS 932 analyser.

Synthesis of 3a:

A solution of bis(di*iso*propylamino)carbene **1** (150 mg; 0.706 mmol) in benzene (10 mL) was added to a solution of $DurBCl_2$ (152 mg; 0.706 mmol) in benzene (5 mL) at room temperature. After 15 min, all volatiles were removed under reduced pressure. The residue was washed with cold pentane (-78 °C) and dried in *vacuo* to afford **3a** as a colorless, crystalline solid (278 mg; 92%). Single crystals suitable for X-ray diffraction were obtained by recrystallization from benzene at room temperature.
^{1}H NMR (500 MHz, C_6D_6): δ = 6.89 (s, 1H, Ar-C*H*), 4.57 (s, 4H, C*H*), 2.66 (s, 6H, Ar-CH_3), 2.21 (s, 6H, Ar-CH_3), 1.06 (s, 24H, CH_3).
^{13}C NMR (126 MHz, C_6D_6): δ = 136.2 (C_q), 133.5 (C_q), 130.4 (CH), 56.67 (CH), 23.14 (CH_3), 21.19 (CH_3), 20.87 (CH_3).
^{11}B NMR (MHz, C_6D_6): δ = 4.8.
Elemental analysis (%) calcd. for $C_{23}H_{41}BN_2Cl_2•0.5C_6H_6$: C 66.96, H 9.51, N 6.01; found C 66.37, H 9.72, N 6.07.

Synthesis of 3b:

A solution of bis(di*iso*propylamino)carbene **1** (35.6 mg; 0.168 mmol) in benzene (3 mL) was added to a solution of $MesBCl_2$ (33.9 mg; 0.168 mmol) in benzene (3 mL) at room temperature. After 15 min, all volatiles were removed under reduced pressure. The residue was washed with hexane and dried *in vacuo* to yield **3b** (64.3 mg; 93%) as a colorless, crystalline solid. Single crystals suitable for X-ray diffraction were obtained by recrystallization from benzene at room temperature.
^{1}**H NMR** (400 MHz, C_6D_6): δ = 6.87 (s, 2H, Ar-C*H*), 4.65 (s, 4H, C*H*), 2.78 (s, 6H, Ar-CH_3), 2.24 (s, 3H, Ar-CH_3), 1.03 (s, 24H, CH_3).
^{13}**C NMR** (126 MHz, C_6D_6): δ = 139.9 (C_q), 134.9 (C_q), 130.5 (CH), 56.71 (CH), 25.57 (CH_3), 23.01 (CH_3), 20.91 (CH_3).
^{11}**B NMR** (160 MHz, C_6D_6): δ = 4.5.
Elemental analysis (%) calcd. for $C_{22}H_{39}BN_2Cl_2$: C 63.94, H 9.51, N 6.78; found C 64.39, H 9.65, N 6.64.

Synthesis of 3c:

A solution of bis(di*iso*propylamino)carbene **1** (50.0 mg; 0.235 mmol) in hexane (7 mL) was added to a solution of $MesBBr_2$ (68.2 mg; 0.0235 mmol) in pentane (7 mL). The colorless precipitate was collected by filtration, washed with pentane, and dried under reduced pressure to afford **3c** (115 mg; 97%) as a colorless, crystalline solid.
^{1}**H NMR** (500 MHz, C_6D_6): δ = 6.86 (s, 2H, Ar-C*H*), 4.80 (s, 4H, C*H*), 2.81 (s, 6H, Ar-CH_3), 2.22 (s, 3H, Ar-CH_3), 1.01 (s, 24H, CH_3).
^{13}**C NMR** (126 MHz, C_6D_6): δ = 139.6 (C_q), 135.1 (C_q), 130.6 (CH), 57.36 (CH), 23.44 (CH_3), 22.99 (CH_3), 20.85 (CH_3).
^{11}**B NMR** (160MHz , C_6D_6): δ = -1.3.
Elemental analysis (%) calcd. for $C_{22}H_{39}BN_2Br_2$: C 52.62, H 7.83, N 5.58; found C 53.18, H 8.10, N 5.16.

Isolation of 4:

The solvent from a benzene solution (3 mL) of **3c** (56.0 mg; 0.193 mmol) was allowed to evaporate over a period of 24 h at room temperature to afford pale yellow crystals. Recrystallization from benzene yielded **4** (46.3 mg; 63%) as colorless crystals.
^{1}**H NMR** (500 MHz, C_6D_6): δ = 4.66 (br s, 2H, C*H*), 3.76 (m, 1H, C*H*), 3.34 (sept, 1H, C*H*, $^{3}J_{HH}$= 6.73 Hz), 2.09 (dd, 1H, CH_2, $^{3}J_{HH}$= 6.56 Hz, $^{2}J_{HH}$= 13.45 Hz), 1.45 (dd, 1H, CH_2, $^{3}J_{HH}$= 7.03 Hz, $^{2}J_{HH}$= 13.45 Hz), 1.13 (d, 3H, CH_3, $^{3}J_{HH}$= 7.13 Hz), 1.11 (d, 9H, CH_3, $^{3}J_{HH}$= 7.13 Hz), 0.89 (d, 3H, CH_3, $^{3}J_{HH}$= 6.35 Hz), 0.87 (d, 3H, CH_3, $^{3}J_{HH}$= 6.73 Hz), 0.82 (d, 3H, CH_3, $^{3}J_{HH}$= 6.73 Hz).
^{13}**C NMR** (126 MHz, C_6D_6): δ = 56.83 (CH), 55.35 (CH), 54.82 (CH), 36.04 (CH_2), 24.79 (CH_3), 24.66 (CH_3), 21.40 (CH_3), 21.39 (CH_3), 20.54 (CH_3).
^{11}**B NMR** (160 MHz, C_6D_6): δ = -3.4.
Elemental analysis (%) calcd. for $C_{13}H_{27}BN_2Br_2$: C 40.88, H 7.12, N 7.33; found C 41.48, H 7.23, N 7.48.

Synthesis of 5a:

KC_8 (139 mg; 0.103 mmol) was added to a solution of **3a** (20.0 mg; 0.0468 mmol) in benzene (8 mL). After stirring for 1 h at room temperature, the reaction mixture was filtered. Slow evaporation of the solvent yielded colorless crystals of **5a** (14.2 mg, 85%), which were washed with cold pentane (-78 °C) and dried in vacuum. Single crystals suitable for X-ray diffraction were obtained by recrystallization from benzene at room temperature.
^{1}H NMR (500 MHz, C_6D_6): δ = 6.88 (s, 1H, Ar-C*H*), 3.10 (m, 3H, C*H*), 2.98 (s, 1H, C*H*), 2.42 (s, 3H, Ar-CH_3), 2.35 (s, 3H, Ar-CH_3), 2.15 (s, 6H, Ar-CH_3), 1.40 (s, 3H, CH_3), 1.29 (s, 3H, CH_3), 1.09 (d, 9H, CH_3, $^3J_{HH}$= 6.71 Hz), 0.98 (d, 3H, CH_3, $^3J_{HH}$= 6.71 Hz), 0.85 (d, 3H, CH_3, $^3J_{HH}$= 5.31 Hz).
^{13}C NMR (126 MHz, C_6D_6): δ = 140.1 (C_q), 134.6 (C_q), 134.4 (C_q), 133.3 (C_q), 132.9 (C_q), 131.4 (CH), 70.42 (C_q), 61.05 (CH), 45.10 (CH), 26.68 (CH_3), 25.90 (CH_3), 24.32 (CH_3), 24.18 (CH_3), 21.31 (CH_3), 21.05 (CH_3), 19.96 (CH_3).
^{11}B NMR (160 MHz, C_6D_6): δ = 45.0.
Elemental analysis (%) calcd. for $C_{23}H_{41}BN_2$: C 77.51, H 11.54, N 7.86; found C 77.81, H 11.52, N 7.46.

Synthesis of 5b:

KC_8 (113 mg; 0.665 mmol) was added to a solution of **3b** (125 mg; 0.302 mmol) in benzene (8 mL). After stirring for 1 h at room temperature, the reaction mixture was filtered. After removal of all volatiles under reduced pressure, **5b** (92.2 mg; 89%) was isolated as a colorless oil. **5b** could also be prepared in an identical manner from **3c**.
^{1}H NMR (500 MHz, C_6D_6): δ = 6.82 (m, 1H, Ar-C*H*), 6.79 (m, 1H, Ar-C*H*), 3.10 (m, 3H, C*H*), 2.93 (s, 1H, C*H*), 2.47 (s, 3H, Ar-CH_3), 2.41 (s, 3H, Ar-CH_3), 2.18 (s, 3H, Ar-CH_3), 1.38 (s, 3H, CH_3), 1.26 (s, 3H, CH_3), 1.08 (d, 6H, CH_3, $^3J_{HH}$= 6.76 Hz), 1.07 (d, 3H, $^3J_{HH}$= 6.76 Hz), 0.95 (d, 3H, CH_3, $^3J_{HH}$= 6.76 Hz), 0.86 (d, 3H, CH_3, $^3J_{HH}$= 5.36 Hz).
^{13}C NMR (126 MHz, C_6D_6): δ = 140.1 (C_q), 134.6 (C_q), 134.4 (C_q), 133.3 (C_q), 132.9 (C_q), 131.4 (CH), 70.42 (C_q), 61.05 (CH), 45.10 (CH), 26.68 (CH_3), 25.90 (CH_3), 24.32 (CH_3), 24.18 (CH_3), 21.31 (CH_3), 21.05 (CH_3), 19.96 (CH_3).
^{11}B NMR (160 MHz, C_6D_6): δ = 45.0.
Elemental analysis (%) calcd. for $C_{22}H_{39}BN_2$: C 77.18, H 11.48, N 8.18; found C 77.29, H 11.61, N 8.06.

Crystallographic methods

The crystal data of **3a**, **3b**, and **5a** were collected on a Bruker X8-APEX II diffractometer with a CCD area detector and multi-layer mirror monochromated Mo_{K_α} radiation. The structure was solved using direct methods, refined with the Shelx software package,[5] and expanded using Fourier techniques. All non-hydrogen atoms were refined anisotropically. Hydrogen atoms were included in structure factors calculations. All hydrogen atoms were assigned to idealised geometric positions.

Crystal data for **3a**: $C_{26}H_{44}BCl_2N_2$, $M_r = 466.34$, colorless block, 0.452×0.302×0.099 mm^3, Monoclinic space group $P2_1/n$, $a = 8.7727(11)$ Å, $b = 16.781(2)$ Å, $c = 18.220(2)$ Å, $\beta = 91.317(5)°$, $V = 2681.6(6)$ Å^3, $Z = 4$, $\rho_{calcd} = 1.155$ g·cm^{-3}, $\mu = 0.258$ mm^{-1}, $F(000) = 1012$, $T = 100(2)$ K, $R_1 = 0.0513$, $wR^2 = 0.1066$, 5472 independent reflections [2θ≤52.74°] and 292 parameters.

Crystal data for **3b**: $C_{22}H_{39}BCl_2N_2$, $M_r = 413.26$, orange block, 0.20×0.15×0.10 mm^3, Monoclinic space group $P2_1/n$, $a = 8.4091(17)$ Å, $b = 23.915(5)$ Å, $c = 13.429(3)$ Å, $\beta = 91.008(7)°$, $V = 2700.2(10)$ Å^3, $Z = 4$, $\rho_{calcd} = 1.017$ g·cm^{-3}, $\mu = 0.249$ mm^{-1}, $F(000) = 896$, $T = 100(2)$ K, $R_1 = 0.0963$, $wR^2 = 0.2453$, 5521 independent reflections [2θ≤52.74°] and 255 parameters.

Crystal data for **5a**: $C_{23}H_{41}BN_2$, $M_r = 356.39$, colorless block, 0.256×0.211×0.054 mm^3, Triclinic space group P-1, $a = 8.8938(17)$ Å, $b = 9.258(2)$ Å, $c = 15.064(3)$ Å, $\alpha = 73.468(12)°$, $\beta = 79.717(9)°$, $\gamma = 77.485(8)°$, $V = 1151.7(5)$ Å^3, $Z = 2$, $\rho_{calcd} = 1.028$ g·cm^{-3}, $\mu = 0.058$ mm^{-1}, $F(000) = 396$, $T = 100(2)$ K, $R_1 = 0.0861$, $wR^2 = 0.1422$, 4534 independent reflections [2θ≤52.042°] and 377 parameters. One of *iso*-propyl group was showing rotational disorder. The coordinates and displacement parameters of methylene atoms of this group were constrained to the same value (EXYZ and EADP keywords). The main four-membered ring was disordered (86:14). The geometry of disordered parts were restrained with SAME keyword. The displacement parameters of atoms belonging to disordered residues were constrained to the same value with similarity restraint SIMU and with 'enhnaced rigid bond' restraint RIGU.

The crystal data of **4** were collected on a Bruker D8-QUEST diffractometer with a CCD area detector and multi-layer mirror monochromated $Mo_{K\alpha}$ radiation. The structure was solved using direct methods, refined with the Shelx software package,[5] and expanded using Fourier techniques. All non-hydrogen atoms were refined anisotropically. Hydrogen atoms were included in structure factors calculations. All hydrogen atoms were assigned to idealised geometric positions.

Crystal data for **4**: $C_{13}H_{28}BBr_2N_2$, $M_r = 383.00$, colorless block, 0.31×0.28×0.28 mm^3, Monoclinic space group $P2_1/n$, $a = 7.1189(6)$ Å, $b = 15.9168(14)$ Å, $c = 15.0043(12)$ Å, $\beta = 91.677(3)°$, $V = 1699.4(2)$ Å^3, $Z = 4$, $\rho_{calcd} = 1.497$ g·cm^{-3}, $\mu = 4.758$ mm^{-1}, $F(000) = 780$, $T = 100(2)$ K, $R_1 = 0.0234$, $wR^2 = 0.0578$, 3473 independent reflections [2θ≤52.74°] and 170 parameters.

Crystallographic data have been deposited with the Cambridge Crystallographic Data Centre as supplementary publication nos. CCDC 1042591 to 1042594. These data can be obtained free of charge from The Cambridge Crystallographic Data Centre via www.ccdc.cam.ac.uk/data_request/cif.

Computational Details

All calculations were carried out in Gaussian 09.[6] Geometry optimizations for all ground states, intermediates, and transitions states were carried out at the B3LYP/6-311G(d) level of theory. All ground states and intermediates were checked by frequency analysis to ensure the existence of no imaginary frequencies. All transition states were located by QST2 calculations, and checked to ensure the existence of only one imaginary frequency. All imaginary frequencies identified for transition states were examined to ensure the continuity of the reaction potential energy surface.

Table S1. Electronic and free energies (kcal/mol) calculated along the proposed reaction pathway. Electronic energies are ZPE corrected.

Compound	E	G
**INT1**	-1035.059990	-1035.122279
**INT2**	-1035.061963	-1035.122623
**INT3**	1035.121643	-1035.184034
**TS1**	-1035.091784	-1035.153595
**5a**	-1035.146750	-1035.207921

Table S2. Cartesian coordinates for **Int1**

B	0.30144900	-0.18155200	-0.11091400
C	-1.09744500	-0.12358900	-0.03788800
H	-1.12689600	2.34639900	-0.42436400
N	-2.16246300	-0.83982000	-0.67158600
C	-3.14756700	-0.00813100	-1.39287500
H	-3.22077500	0.90045700	-0.79330700
C	-4.54814800	-0.63512100	-1.39895100
H	-4.61065000	-1.51991000	-2.03916500
H	-5.28056000	0.08299100	-1.78022600
H	-4.84907000	-0.92575400	-0.39069800
C	-2.72635000	0.41467100	-2.81240200
H	-2.73300100	-0.42941100	-3.50862900
H	-1.72217600	0.84575000	-2.80997200
H	-3.41277300	1.16894000	-3.21111800
C	-1.86293300	-2.16475600	-1.24198800
H	-2.73064500	-2.42088200	-1.85545800
C	-1.77514200	-3.23059700	-0.14039900
H	-1.60132800	-4.22258600	-0.56926400
H	-2.70123200	-3.26546400	0.43733800
H	-0.95467800	-3.01746100	0.54722800
C	-0.63500200	-2.24333700	-2.17202400
H	-0.64855800	-1.46411500	-2.93548900
H	-0.61204900	-3.21105900	-2.68406500
H	0.30373100	-2.15680000	-1.61682500
C	-1.33442500	2.30971100	0.64691600
C	-2.63828600	3.09377100	0.89009200
H	-3.48524400	2.65157500	0.36191200
H	-2.52500100	4.12204600	0.53379600
H	-2.89612300	3.14874600	1.95129800

C	-0.16416400	2.99825900	1.37015900
H	-0.08751600	4.05108600	1.07941200
H	0.78164900	2.50879800	1.12959700
H	-0.29055600	2.96933700	2.45698700
C	-0.99791800	-0.48751300	3.03174800
H	-0.99504500	-1.46060200	2.53877600
H	-1.33823800	-0.63531900	4.06124200
H	0.02879100	-0.11637100	3.06102300
C	-3.36778500	-0.01872200	2.30764400
H	-4.04729100	0.70086800	1.84636400
H	-3.71232400	-0.19512300	3.33241700
H	-3.44233700	-0.95414600	1.75270100
C	1.82691900	-0.12154900	-0.14308000
C	2.61564000	-0.96303100	0.69213400
C	4.01452600	-0.85028300	0.68433000
C	4.60886100	0.07347900	-0.17270200
H	5.69353300	0.15467600	-0.17838300
C	3.87473200	0.88873000	-1.03239000
C	2.47456000	0.79598000	-1.02150900
C	1.98322500	-1.99759100	1.58810000
H	2.41459300	-2.98876400	1.41424200
H	0.91041500	-2.06157400	1.42304400
H	2.14321600	-1.77005100	2.64794700
C	4.87945900	-1.71259100	1.57265100
H	5.93428300	-1.45101100	1.46790800
H	4.78004100	-2.77654300	1.33247700
H	4.61575000	-1.60540500	2.62962200
C	4.59362000	1.84927800	-1.94970800
H	4.37563300	1.64878700	-3.00390700
H	5.67551400	1.78189200	-1.81942600
H	4.30372800	2.88870200	-1.76354100
C	1.69078400	1.67413000	-1.96612300
H	1.96539500	1.47790900	-3.00832300
H	1.88961600	2.73640800	-1.78703400
H	0.61969300	1.50867400	-1.86540400
C	-1.91792400	0.49850300	2.30725500
H	-1.90285000	1.42389700	2.89183100
N	-1.42486900	0.86838500	0.96416600

Table S3. Cartesian coordinates for `Int2`

B	-0.16832300	-0.03606300	-0.00727300
C	1.18780200	0.17254300	-0.16188700
H	1.05498400	-2.36138900	-0.57133200
N	2.41134100	0.68651600	-0.56190900
C	3.21578700	-0.15565000	-1.48680000
H	2.96530400	-1.18171000	-1.21581700
C	4.71916600	0.01655300	-1.23744000
H	5.07715800	1.01008100	-1.52206700
H	5.28985500	-0.70966500	-1.82401200
H	4.95631600	-0.13260900	-0.18152600
C	2.86798900	0.00143200	-2.97610600
H	3.15741100	0.98142200	-3.36542300
H	1.79638900	-0.13208400	-3.14107300
H	3.39518900	-0.75171200	-3.57042600
C	2.46998600	2.15143800	-0.80121900
H	3.39519000	2.31545800	-1.36022300

C	2.59886500	2.93795300	0.50678400
H	2.70390700	4.00764800	0.30136300
H	3.47196700	2.61349000	1.07670600
H	1.71189400	2.80914100	1.13150800
C	1.31644400	2.70950700	-1.65340100
H	1.21452800	2.17884800	-2.60056600
H	1.49271100	3.76624700	-1.87869900
H	0.36306500	2.63554400	-1.12822800
C	1.12338700	-2.37071300	0.51751200
C	2.50769900	-2.91408600	0.89900700
H	3.31442400	-2.26249900	0.56191400
H	2.65449400	-3.89348500	0.43393300
H	2.61779500	-3.05299700	1.97758900
C	0.01698600	-3.28763100	1.05498600
H	0.09202200	-4.27572400	0.59171800
H	-0.97328600	-2.88630700	0.83779000
H	0.09109400	-3.43421300	2.13620900
C	-0.11824800	-0.04064400	3.05727200
H	-0.18405300	1.01323900	2.79039900
H	-0.01153500	-0.09641700	4.14485700
H	-1.05949900	-0.52190600	2.78497800
C	2.38520300	-0.00733700	2.73778000
H	3.24987800	-0.49806800	2.28959800
H	2.52483000	0.00112300	3.82355000
H	2.37374800	1.02477500	2.39374700
C	-1.71829200	0.09166300	-0.19379800
C	-2.43749100	1.17459700	0.37322500
C	-3.83880200	1.22924700	0.27021600
C	-4.50599400	0.21797800	-0.41510000
H	-5.59098700	0.25575100	-0.48250700
C	-3.82795200	-0.82020200	-1.04793400
C	-2.42668000	-0.87478300	-0.95412700
C	-1.73562400	2.32356800	1.05934600
H	-1.99320400	3.27690700	0.58535600
H	-0.65406000	2.21735200	1.01212600
H	-2.02291100	2.41595300	2.11269300
C	-4.62780400	2.36357200	0.88068300
H	-5.70055300	2.21348000	0.74260800
H	-4.37159000	3.32955800	0.43173700
H	-4.44420500	2.46218600	1.95576900
C	-4.60507000	-1.85119600	-1.83242300
H	-4.38267700	-1.80424700	-2.90450600
H	-5.68064800	-1.70205300	-1.71687000
H	-4.37655700	-2.87272900	-1.51172900
C	-1.70554600	-1.94912800	-1.73720400
H	-2.03749900	-1.95873200	-2.78043000
H	-1.89170600	-2.95577500	-1.34655900
H	-0.62983500	-1.78193400	-1.74314400
C	1.07727800	-0.72198200	2.39576100
H	1.13500700	-1.72442900	2.82562100
N	0.89047900	-0.93471600	0.90604300

Table S4. Cartesian coordinates for **Int3**

B	0.19687100	0.61102000	0.11088300
C	1.23196500	-0.38603300	0.52330000

H	2.48096800	1.52597100	0.12991500
N	2.01537900	-1.29901900	0.08064900
C	2.25542200	-1.58047000	-1.38327200
H	1.66190600	-0.81724100	-1.88451300
C	1.71787100	-2.95536600	-1.78578600
H	2.27754600	-3.77320400	-1.32372900
H	1.79488600	-3.08152800	-2.86968300
H	0.66694200	-3.05864100	-1.50805400
C	3.72251700	-1.38206400	-1.77240500
H	4.38178200	-2.12851900	-1.32172400
H	4.07763300	-0.39110700	-1.47973700
H	3.83090800	-1.46763700	-2.85735900
C	2.75409000	-2.19841500	1.05031400
H	3.32764200	-2.90533800	0.44676600
C	1.75642900	-2.97772100	1.90377400
H	2.28260500	-3.63011200	2.60637100
H	1.10208400	-3.59816100	1.28658600
H	1.13158400	-2.28366100	2.46881000
C	3.72267900	-1.37641400	1.89746400
H	4.46538300	-0.86734800	1.27751100
H	4.25527800	-2.01984800	2.60301800
H	3.17181100	-0.62184300	2.46294100
C	1.89618300	2.44485800	0.04091300
C	2.37083500	3.14709100	-1.24098700
H	2.19679000	2.51652500	-2.11659700
H	3.44241100	3.36367100	-1.18646700
H	1.85811100	4.09881000	-1.40776800
C	2.21038700	3.28062500	1.29283500
H	3.28373200	3.48480800	1.35606900
H	1.91547700	2.74308200	2.19672000
H	1.69789600	4.24685100	1.28990700
N	0.48422600	2.01104300	-0.02900000
C	-1.41366900	3.21439600	1.12452100
H	-2.11017600	2.37687800	1.17471400
H	-2.00326000	4.13637900	1.08510900
H	-0.82885500	3.22680300	2.04671200
C	-1.33122000	3.10829800	-1.40030200
H	-0.68755500	3.05400300	-2.28132100
H	-1.92241400	4.02728000	-1.47139100
H	-2.02356700	2.26636800	-1.42902800
C	-1.26356400	-0.05935300	0.05212000
C	-1.81099300	-0.44106400	-1.19545800
C	-3.05306400	-1.09652400	-1.25905900
C	-3.73268300	-1.36543400	-0.07327000
H	-4.69506100	-1.87017300	-0.12222000
C	-3.21821800	-1.01483800	1.17199600
C	-1.97090700	-0.36552400	1.23851100
C	-1.08092500	-0.16554300	-2.49305700
H	-0.78396100	-1.09463000	-2.99411300
H	-0.18447200	0.43246500	-2.33664500
H	-1.70928500	0.37934000	-3.20510900
C	-3.65730400	-1.51221600	-2.57998100
H	-4.60187200	-2.03943700	-2.43032000
H	-2.99516900	-2.17741300	-3.14486800
H	-3.86095100	-0.65294200	-3.22855900
C	-3.99990000	-1.34101800	2.42269400
H	-3.44263200	-2.00342400	3.09390200

H	-4.94210800	-1.83706800	2.17970100
H	-4.24003600	-0.44345700	3.00252100
C	-1.42947500	-0.00016800	2.60415800
H	-1.31057800	-0.88775600	3.23568700
H	-2.11018000	0.67104800	3.14036000
H	-0.45583600	0.47928000	2.54213200
C	-0.50317900	3.11381800	-0.10763600
H	0.08333600	4.03612600	-0.13052600

Table S5. Cartesian coordinates for **TS1**

B	-0.16181800	0.64508300	-0.10124600
C	-1.45766900	-0.10637200	-0.37614200
H	-2.21403500	0.90356900	-0.51393000
N	-1.98350700	-1.25457500	0.03500700
C	-1.48417200	-1.99864300	1.22482300
H	-0.71955900	-1.34678300	1.64534600
C	-0.80957000	-3.31751800	0.82946100
H	-1.51461000	-4.02602600	0.38470700
H	-0.37907400	-3.79871900	1.71237300
H	-0.00237900	-3.13882600	0.11688200
C	-2.57070900	-2.20330900	2.28899000
H	-3.36785200	-2.86956700	1.94791500
H	-3.02248800	-1.25201800	2.57822700
H	-2.13427100	-2.65488900	3.18464000
C	-3.10074600	-1.87617800	-0.73366000
H	-3.30028700	-2.84049800	-0.26042600
C	-2.69381300	-2.13744700	-2.18573600
H	-3.50773600	-2.62571600	-2.72963300
H	-1.81329800	-2.78193900	-2.23794100
H	-2.45472000	-1.19905600	-2.68977300
C	-4.38168300	-1.03990400	-0.64505200
H	-4.67679600	-0.87079200	0.39323900
H	-5.20487400	-1.55171900	-1.15168100
H	-4.24805300	-0.06794700	-1.12531500
C	-1.92897300	2.15014300	0.20766800
C	-2.45659200	1.98976200	1.62214400
H	-1.87955800	1.26020000	2.19011900
H	-3.50303200	1.66449300	1.62247600
H	-2.42190700	2.94465000	2.16893000
C	-2.67740500	3.21031700	-0.58033800
H	-3.74513300	2.97157000	-0.59867800
H	-2.33270600	3.27198900	-1.61536100
H	-2.59557900	4.21187900	-0.13501400
N	-0.52076200	2.03934500	0.00551800
C	0.97460700	3.16191700	-1.66663000
H	1.72134300	2.36637500	-1.69826400
H	1.47987900	4.10567000	-1.89489700
H	0.24249500	2.97068600	-2.45496600
C	1.32425200	3.51422400	0.81622800
H	0.83808000	3.58031500	1.79258700
H	1.83744400	4.46275200	0.62892100
H	2.07967400	2.72782300	0.86261900
C	1.29505300	0.00269100	-0.08674700
C	2.01233700	-0.12485200	1.12749300
C	3.28252200	-0.72741900	1.14806700

C	3.82356500	-1.19815400	-0.04563200
H	4.80788000	-1.66103800	-0.03021300
C	3.14410700	-1.09880300	-1.25712200
C	1.86970300	-0.50244800	-1.27883800
C	1.43049100	0.35867600	2.43934700
H	1.27913600	-0.46875000	3.14301300
H	0.47337900	0.85862800	2.30260900
H	2.09429700	1.06923400	2.94318000
C	4.06310000	-0.87577700	2.43287700
H	5.01698100	-1.37712700	2.25624600
H	3.51767000	-1.46127200	3.18111200
H	4.28239000	0.09254300	2.89606200
C	3.78362300	-1.63092900	-2.51794300
H	3.18305100	-2.42081400	-2.98207300
H	4.77157600	-2.04826000	-2.31188400
H	3.90759100	-0.85043700	-3.27630700
C	1.14859000	-0.41238500	-2.60713600
H	0.99367100	-1.40402700	-3.04715700
H	1.72473300	0.16269900	-3.34124400
H	0.16996900	0.05343100	-2.50903400
C	0.29987500	3.23487700	-0.29207000
H	-0.38730000	4.08410100	-0.30916300

Table S6. Cartesian coordinates for **5a**

B	-0.04089100	0.61390500	-0.23378300
C	-1.53239600	0.01400100	-0.47103600
H	-1.60739200	-0.22188700	-1.53810200
N	-2.19928600	-1.02817400	0.28155200
C	-1.59615600	-1.47811600	1.54517800
H	-0.99276000	-0.63468600	1.88878600
C	-0.64910400	-2.68807700	1.42659200
H	-1.18740900	-3.59722700	1.14198700
H	-0.16163600	-2.89011500	2.38595300
H	0.13393700	-2.50569000	0.68862500
C	-2.65637300	-1.72360000	2.63087300
H	-3.29552800	-2.58014600	2.39688400
H	-3.29879800	-0.84867700	2.74898600
H	-2.18001100	-1.93457400	3.59331700
C	-3.00290200	-2.01070200	-0.46139000
H	-3.28296900	-2.77617400	0.26732600
C	-2.26727100	-2.74214500	-1.60551000
H	-2.88314600	-3.56327700	-1.98597800
H	-1.31704300	-3.16182100	-1.27192100
H	-2.06277900	-2.08044400	-2.45206900
C	-4.31262700	-1.39953700	-0.98133500
H	-4.88302600	-0.95226000	-0.16429800
H	-4.93706600	-2.16029500	-1.46071300
H	-4.11965600	-0.62138700	-1.72514000
C	-1.98905400	1.53869900	-0.32492200
C	-2.78589500	1.83119900	0.94273100
H	-2.25821900	1.49278500	1.83644000
H	-3.74577200	1.31093300	0.90881000
H	-2.98787600	2.90163800	1.05136500
C	-2.69363100	2.12152800	-1.55171800
H	-3.68907200	1.68366100	-1.66179000

H	-2.13052200	1.91675100	-2.46718400
H	-2.82908900	3.20581200	-1.47680500
N	-0.54525700	1.92559000	-0.24064000
C	1.31312700	3.35175500	-1.04660300
H	2.08599000	2.78726000	-0.52085700
H	1.65226000	4.38789500	-1.13403400
H	1.22305400	2.93860100	-2.05393300
C	0.08678500	3.94312400	1.08526000
H	-0.86156500	3.90584200	1.62403500
H	0.37936100	4.99360100	0.99309700
H	0.84125700	3.43775800	1.69302800
C	1.42278100	0.02934700	-0.15186800
C	2.13086400	-0.01830100	1.07010800
C	3.40567900	-0.60979900	1.12715700
C	3.95420600	-1.14083400	-0.03726700
H	4.93852000	-1.60121200	0.00901800
C	3.28863100	-1.09764300	-1.26076500
C	2.01590100	-0.50580500	-1.31909200
C	1.54663000	0.54973600	2.34546600
H	1.41494000	-0.22350900	3.11064900
H	0.57790700	1.01977500	2.18305700
H	2.20252400	1.30869700	2.78610200
C	4.18201900	-0.68085000	2.42081600
H	5.14088200	-1.18248600	2.27588900
H	3.63763000	-1.22931800	3.19706400
H	4.39062900	0.31351400	2.83056600
C	3.94319900	-1.68007900	-2.49112900
H	3.32159300	-2.44783100	-2.96352600
H	4.90257200	-2.13975800	-2.24511400
H	4.13247900	-0.91803600	-3.25536400
C	1.30841700	-0.44339300	-2.65639300
H	1.95693200	-0.02881400	-3.43458600
H	0.41432700	0.17810700	-2.62239200
H	1.00172600	-1.43726000	-3.00251200
C	-0.02464600	3.29371200	-0.30339800
H	-0.75003200	3.87748700	-0.88265100

References

[1] R. W. Alder, P. R. Allen, M. Murray and A. G. Orpen, *Angew. Chem. Int. Ed.*, 1996, **35**, 1121.

[2] A. Eckert, H. Pritzkow and W. Siebert, *Eur. J. Inorg. Chem.,* 2002, 2064.

[3] W. Schacht and D. Kaufmann, *Chem. Ber.*, 1987, **120**, 1331.

[4] H. Braunschweig, Q. Ye and K. Radacki, *Chem. Commun.*, 2012, **48**, 2701.

[5] G. Sheldrick, *Acta Cryst.*, 2008, **A64**, 112.

[6] Gaussian 09, Revision **D.01**, M. J. Frisch, G. W. Trucks, H. B. Schlegel, G. E. Scuseria, M. A. Robb, J. R. Cheeseman, G. Scalmani, V. Barone, B. Mennucci, G. A. Petersson, H. Nakatsuji, M. Caricato, X. Li, H. P. Hratchian, A. F. Izmaylov, J. Bloino, G. Zheng, J. L. Sonnenberg, M. Hada, M. Ehara, K. Toyota, R. Fukuda, J. Hasegawa, M. Ishida, T. Nakajima, Y. Honda, O. Kitao, H. Nakai, T. Vreven, J. A. Montgomery, Jr., J. E. Peralta, F. Ogliaro, M. Bearpark, J. J. Heyd, E. Brothers, K. N. Kudin, V. N. Staroverov, R.

Kobayashi, J. Normand, K. Raghavachari, A. Rendell, J. C. Burant, S. S. Iyengar, J. Tomasi, M. Cossi, N. Rega, J. M. Millam, M. Klene, J. E. Knox, J. B. Cross, V. Bakken, C. Adamo, J. Jaramillo, R. Gomperts, R. E. Stratmann, O. Yazyev, A. J. Austin, R. Cammi, C. Pomelli, J. W. Ochterski, R. L. Martin, K. Morokuma, V. G. Zakrzewski, G. A. Voth, P. Salvador, J. J. Dannenberg, S. Dapprich, A. D. Daniels, Ö. Farkas, J. B. Foresman, J. V. Ortiz, J. Cioslowski, and D. J. Fox, Gaussian, Inc., Wallingford CT, 2009.
